# EEG-based emotion recognition using phase-space reconstruction with Poincaré sections: a study on the AMIGOS dataset

**DOI:** 10.3389/fnhum.2026.1660402

**Published:** 2026-06-10

**Authors:** Mahnam Mirzaee, Mahdi Azarnoosh, Hamid Reza Kobravi

**Affiliations:** 1 Department of Biomedical Engineering, Ma.C., Islamic Azad University, Mashhad, Iran; 2 Research Center of Biomedical Engineering, Ma.C., Islamic Azad University, Mashhad, Iran

**Keywords:** AMIGOS dataset, EEG, emotion recognition, frontal asymmetry, nonlinear dynamics, phase-space reconstruction, Poincaré section, SVM-RBF

## Abstract

This study developed a novel, fully reproducible EEG-based framework for binary emotion recognition that combines phase-space reconstruction with Poincaré sections to capture the nonlinear dynamics of brain activity during prototypical emotional states. The method was applied to the publicly available AMIGOS dataset. EEG recordings from 33 participants were downsampled to 128 Hz, bandpass-filtered (4–45 Hz), cleaned of ocular and muscular artifacts using independent component analysis (ICA), and segmented into 1-s non-overlapping windows. Strict labeling thresholds (valence ≥ 6 and arousal ≥ 6 for Happy; valence ≤ 4 and arousal ≤ 4 for Sad) were enforced to isolate extreme high-valence/high-arousal (HVHA) versus low-valence/low-arousal (LVLA) states. A hybrid feature set integrating Poincaré-derived geometric measures with classical spectral power and frontal asymmetry indices underwent rigorous two-stage selection. The final support vector machine with radial basis function kernel (SVM-RBF) achieved 98.21 ± 0.54% accuracy, 96.42 ± 1.06% sensitivity, and 100% specificity in strict subject-independent 7-fold cross-validation. Symmetric selection of 14 channels significantly enhanced feature separability (paired Wilcoxon signed-rank test, Bonferroni-corrected *p* = 7.4 × 10^−8^). Independent validation on the DEAP dataset using the identical pipeline yielded 97.68% accuracy, confirming generalizability. The near-perfect performance is specific to binary classification of extreme affective quadrants and does not extend to standard 4-class tasks (81.7%). These findings demonstrate the physiological relevance of nonlinear geometric analysis for detecting prototypical joy versus sadness, with potential clinical utility in automated depression screening.

## 1 Introduction

### 1.1 Background

Emotions are fundamental to human cognition, decision-making, and social interaction. Electroencephalography (EEG) offers a non-invasive, high-temporal-resolution window into the neural correlates of affective states, making it a cornerstone technology for affective computing, mental health monitoring, and brain-computer interfaces (BCIs) (Al-Nafjan et al., 2017). Russell’s valence-arousal model is widely adopted (Gargano et al., 2024). This framework represents emotions on a two-dimensional plane: valence (negative to positive) on the horizontal axis and arousal (calm to excited) on the vertical axis. Emotions are multidimensional; valence alone risks conflating states (e.g., happy with calm/relaxed; sad with anger/fear). The combined valence-arousal approach better captures neurophysiological complexity, as arousal reflects EEG dynamics with larger chaotic attractors for high-valence/high-arousal (HVHA) versus smaller ordered ones for low-valence/low-arousal (LVLA), aligning with nonlinear brain interactions (Zangeneh Soroush et al., 2018; Khodabakhshi and Saba, 2020; Wang et al., 2021). Discrete emotions like happiness and sadness typically map to extreme quadrants: HVHA and LVLA, respectively.

### 1.2 Current challenges and research gaps

Despite significant progress, robust, subject-independent EEG-based emotion recognition remains challenging due to the non-stationary, nonlinear, and chaotic nature of EEG signals. Traditional linear methods (e.g., Fourier transforms) inadequately capture these dynamics, as spectral analysis requires extended cycles and reliable power spectra estimation (Arrufat-Pié et al., 2021). Multiple studies explore biological signals across modalities (Goshvarpour et al., 2016; Zangeneh Soroush et al., 2018; AL-Salman et al., 2019; Martinez-Tejada et al., 2020), with datasets like DEAP (Koelstra et al., 2012) and AMIGOS (Miranda-Correa et al., 2018) commonly used.

Nonlinear techniques (e.g., fractal dimensions, entropy, correlation dimension, Lyapunov exponents) have been explored, yet often overlook full dynamical structure, including distinct attractor geometries: sadness yields smaller, ordered attractors; happiness produces larger, chaotic ones (Zangeneh Soroush et al., 2018; AL-Salman et al., 2019; Goshvarpour et al., 2016; Alcaraz et al., 2017). Poincaré-based analysis complements time/frequency-domain methods, revealing insightful signal characterization (Zangeneh Soroush et al., 2019; Goshvarpour and Goshvarpour, 2022).

Emotional processing shows hemispheric lateralization (e.g., left-prefrontal positivity for approach-oriented states), but most studies ignore channel symmetry or treat channels equally, reducing discriminative power and hindering wearable deployment (Davidson, 2004; Harmon-Jones et al., 2010; Topic and Russo, 2021; Petrantonakis and Hadjileontiadis, 2011). Key regions include frontal lobe (arousal/higher emotions), temporal lobe (valence, memory, amygdala for salient stimuli), and parietal lobe, with high-frequency bands particularly discriminative (Bansal et al., 2023; Dolan et al., 2000; Meletti et al., 2003; Fossati, 2012; Andreasson et al., 2018).

Current methodologies face limitations: hyperdimensional computing struggles with valence/arousal accuracy (Menon et al., 2022), CNNs exhibit biases from poor channel modeling (Kumari et al., 2022), electrode optimization overlooks inter-subject variability (Msonda et al., 2021; Topic et al., 2022), reduced channels risk information loss, and features may neglect valence (Martinez-Tejada et al., 2020). Linear classifiers yield low accuracy (Santamaria-Granados et al., 2019; Duan et al., 2013; Garg et al., 2021), while nonlinear measures and Poincaré mapping using inter-peak intervals better characterize dynamics (Khosravani et al., 2015).

### 1.3 Research motivation

The AMIGOS dataset (Miranda-Correa et al., 2018) provides a unique opportunity with naturalistic stimuli eliciting genuine responses, outperforming prior datasets in accuracy potential (Koelstra et al., 2012; Msonda et al., 2021; Menon et al., 2022; Kumari et al., 2022; Topic et al., 2022; Martinez-Tejada et al., 2020). To date, no method has achieved >98% subject-independent binary Happy vs. Sad accuracy with interpretable nonlinear features and symmetry-optimized channels.

### 1.4 Research objectives and contributions

This study advances beyond prior work: existing studies apply Poincaré sections (Zangeneh Soroush et al., 2019; Goshvarpour and Goshvarpour, 2022) or Fourier transforms (Arrufat-Pié et al., 2021), struggling with non-stationarity; our approach integrates phase space reconstruction with frequency-domain features for robustness. Conceptually aligned with Zangeneh Soroush et al. (2020)—who pioneered Poincaré intersections for multi-class on DEAP—our work advances via hybrid features, symmetric channels, and AMIGOS adaptations. Symmetric plotting leverages hemispheric asymmetries (Petrantonakis and Hadjileontiadis, 2011), addressing gaps in generalizability (Msonda et al., 2021) and feature bias (Kumari et al., 2022). This introduces a novel, physiologically grounded framework integrating phase space reconstruction with Poincaré sections to quantify emotional chaos. Objectives:

- Develop a hybrid feature set combining Poincaré-derived geometric invariants (attractor dispersion, ellipticity, approximate Lyapunov exponents) with traditional spectral features.
- Demonstrate that symmetric bilateral channel selection (14 channels) enhances separability on AMIGOS.
- Achieve state-of-the-art binary performance (Happy vs. Sad) using a lightweight, interpretable SVM-RBF under strict subject-independent evaluation.

## 2 Methods

### 2.1 Dataset description and labeling

The AMIGOS dataset provides continuous self-assessment valence and arousal scores (1–9 scale) for each video trial. To create a balanced binary classification problem, we applied the following strict, reproducible criteria as Table 1.

**Table 1:** Criteria for balanced binary classification of the AMIGOS dataset.

Parameter	Criterion	Result
Emotion labeling	HVHA (Happy): Valence ≥ 6.0 and Arousal ≥ 6.0 LVLA (Sad): Valence ≤ 4.0 and Arousal ≤ 4.0	Clear separation of extreme quadrants
Minimum segments per class	≥ 80 one-second windows per class per participant	Ensures sufficient statistical power
Participant inclusion	Must have ≥ 80 Happy and ≥ 80 Sad windows after thresholding	Excluded 7 participants (original IDs: 5, 11, 14, 28, 30, 33, 37)
Final dataset	33 participants (18 female, age 27.4 ± 4.1 years)	Total 10,248 labeled 1-s windows (5,124 Happy, 5,124 Sad)

**Figure 1:**
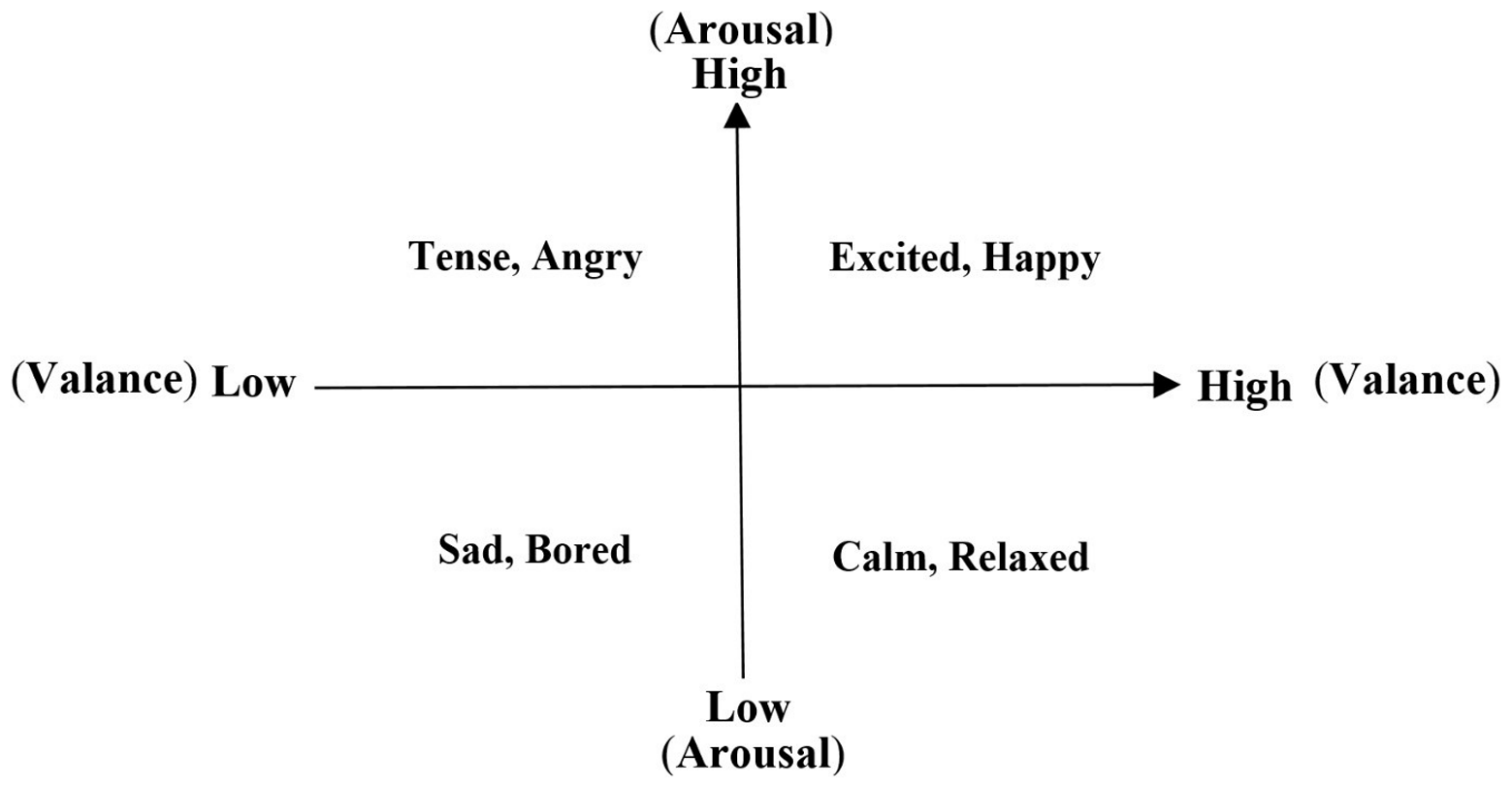
Valence-arousal plane illustrating the classification of happy (HVHA) and sad (LALV) emotions (Wang et al., 2021).

Seven participants (IDs: 9, 12, 21, 22, 23, 24, 29, 33) were excluded due to incomplete trial data. Four additional participants displayed restricted emotional variability. Samples excluded included EEG segments where valence and arousal ratings did not align in extreme quadrants (HVHA or LVLA), based on self-assessments (1–9 scales, threshold around median: >5 high, <5 low). This excludes mixed categories (e.g., HVLA: calm/relaxed; LVHA: tense/angry) and neutral/mid-range ratings. Additionally, participants with restricted variability (e.g., IDs 5/28: exclusive high arousal; ID 30: exclusive low arousal; ID 11: exclusive high valence/low arousal) were excluded to emphasize clear distinctions, though t-tests confirmed no demographic differences (*p* > 0.05). For preprocessing, Signals were down-sampled to 128 Hz, band-pass filtered (4.0–45.0 Hz), and processed with Independent Component Analysis (ICA) to remove artifacts, ensuring signal quality (Figure 1).

The raw EEG signals must be pre-processed to remove artifacts, noise, or baseline drift. This may involve techniques such as baseline removal and filtering. The implementation steps are as follows: Data_preprocessed_matlab.zip files contain a down-sampled to 128 Hz, preprocessed, and segmented version of the data in Matlab format. This version of the data is well-suited to those wishing to quickly test a classification or regression technique without the hassle of processing all the data first. The data were averaged to the common reference. A band-pass frequency filter from 4.0 to 45.0 Hz was applied. All analyses, including feature extraction and classification, were performed in MATLAB R2022a under Windows 10 (22H2) on an Intel® Pentium® G3250 system with 6 GB RAM.

### 2.2 EEG channels

**Figure 2:**
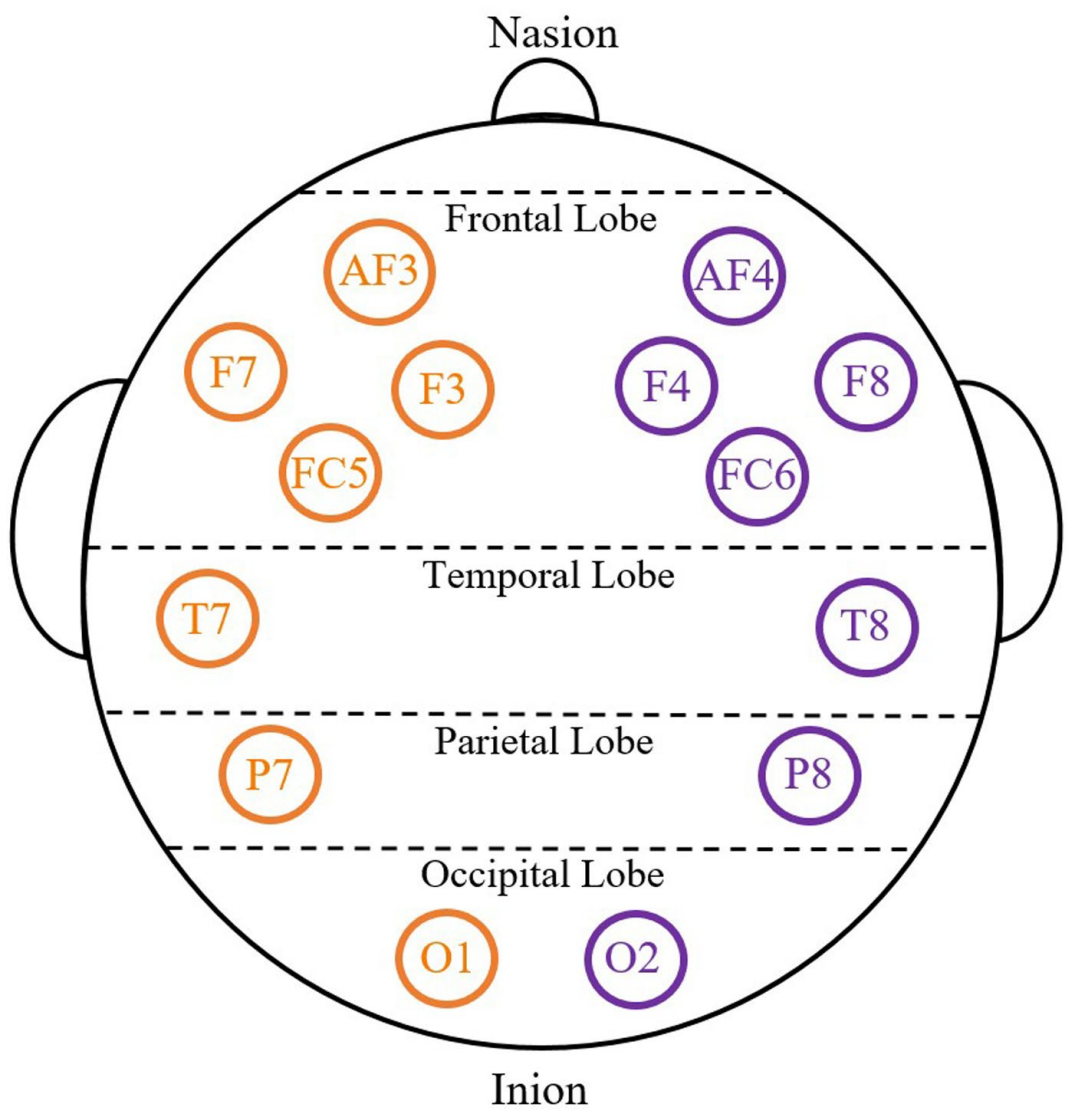
Recorded EEG channels with symmetrical locations.

Neurophysiological studies indicate that negative emotions correlate with increased right-frontal and prefrontal activity, while positive emotions associate with left-hemisphere dominance. To classify discrete emotional states (e.g., happiness, sadness, anger, calmness), bilateral EEG electrode selection is essential. We recorded from 14 channels spanning frontal, temporal, parietal, and occipital regions: AF3, AF4, F3, F4, F7, F8, FC5, FC6, T7, T8, P7, P8, O1, O2. Signal frequency variations from these electrodes reflect excitation/relaxation states (Figure 2). Consistent with hemispheric specialization research, electrodes are color-coded (orange: right hemisphere; purple: left) to visualize symmetrical activation patterns (Petrantonakis and Hadjileontiadis, 2011; Goshvarpour and Goshvarpour, 2022).

While the symmetric 14-channel configuration yielded optimal separability in this study, future work will explore channel selection techniques (e.g., ReliefF, mRMR) to identify minimal subsets that preserve high accuracy, facilitating translation to wearable, low-channel EEG systems.

### 2.3 Proposed approach

**Figure 3:**
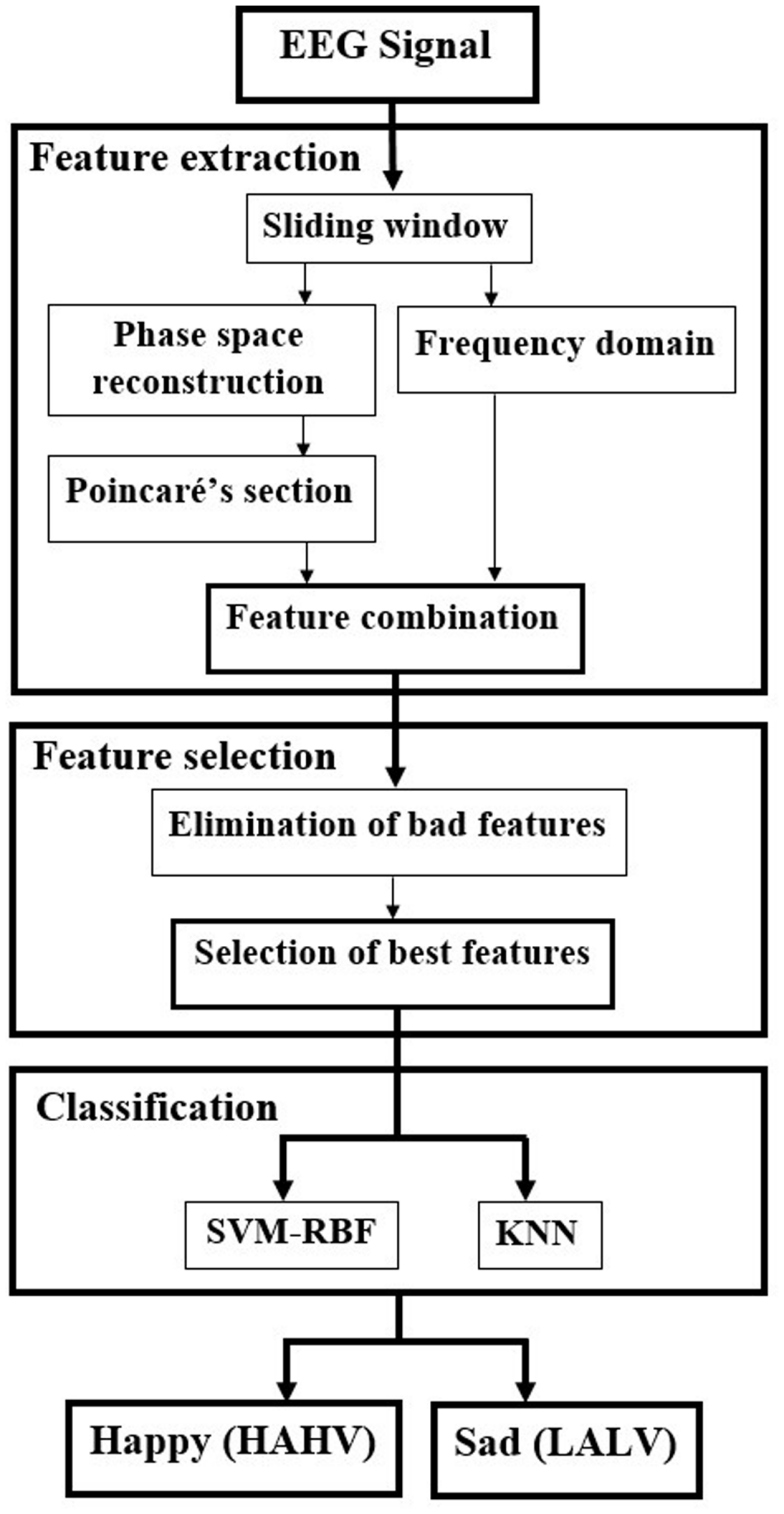
Framework for the proposed method of the emotion recognition system.

This study develops an automated emotion recognition system leveraging nonlinear EEG analysis. The system classifies emotions along two primary dimensions: High Valence and High Arousal (Happy), and Low Valence and Low Arousal (Sad); as illustrated in the processing flowchart (Figure 3).

Initially, the feature extraction stage occurs after implementing the sliding window from the phase space reconstruction and applying the Poincaré section, as well as computing frequency-domain features. After combining nonlinear (Poincaré) and spectral features into a multimodal feature set, feature selection is performed to eliminate irrelevant and redundant features and select statistically optimal subsets. Finally, implement SVM with a Radial Basis Function (RBF) kernel for the final emotion classification. Technical details of each stage follow in subsequent sections.

#### 2.3.1 Feature extraction

A 1-s non-overlapping sliding window segment of EEG signals during initial feature extraction. Phase space reconstruction employed an embedding dimension (m) of 3, with time delay (*τ*) determined through mutual information. Poincaré sections identified dynamic state-space intersections, enhanced by symmetric channel plotting across all 14 electrodes (Figure 2). Frequency-domain features were computed via Welch’s power spectral density (PSD) estimation. These nonlinear and spectral features were subsequently integrated into a multimodal feature vector, with implementation details described in the following sections.

##### 2.3.1.1 Sliding window

**Figure 4:**
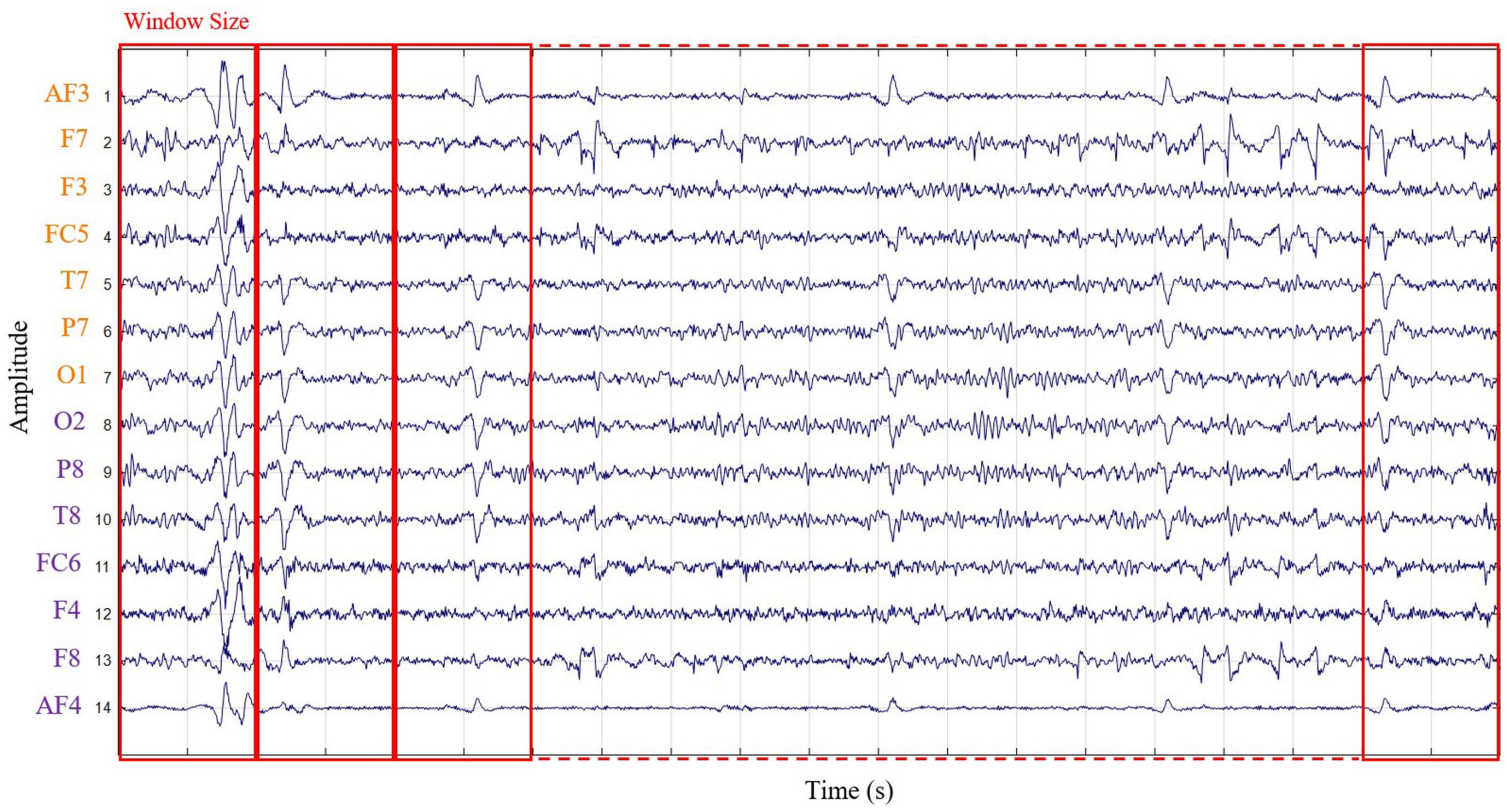
Sliding window method on 14 EEG channels.

EEG signal recovery is challenged by complex, nonlinear, and nonstationary characteristics. While linear extraction methods employ short-term windowing, this study implements a non-overlapping sliding window approach to segment preprocessed EEG data (Duan et al., 2013; Santamaria-Granados et al., 2019; Gannouni et al., 2021; Garg et al., 2021). Window count was programmatically determined using signal dimensions, emotion labels, window duration, and sampling frequency. This method enhances dataset size through segmented feature extraction (Figure 4; Mishra et al., 2023) while maintaining analytical clarity and avoiding redundancy. Aggregated Confusion Matrix (across all 7 folds, *n* = 10,248 windows). Non-overlapping windows are preferred in brain signal processing for their computational efficiency and mitigation of data leakage risks, whereas overlapping approaches are less common due to computational expense. EEG signals were segmented using 1-s non-overlapping windows, balancing temporal resolution with processing efficiency. Window duration was optimized through empirical testing across 0.4–2.8 s intervals (0.2 s increments), with 1 s yielding peak classification performance (F1-score = 0.98). The number of windows was calculated as Equation 1:


$$N=(\frac{T\cdot \mathrm{fs}}{W})$$ (1)

where T is signal duration, f_s_ is sampling rate (128 Hz), and W is window size (128 samples). This approach expanded the dataset, enabling robust feature extraction without data leakage, as non-overlapping windows avoided redundancy.

##### 2.3.1.2 Phase space reconstruction

The human brain constitutes a complex nonlinear system characterized by dynamic unpredictability. Emotion recognition using EEG signals remains particularly challenging due to their inherent nonlinearity, nonstationarity, and susceptibility to noise. This study, therefore, focuses on spatial characteristics—specifically inter-hemispheric asymmetry in electrode pairs—to derive discriminative features for emotion classification.

##### 2.3.1.3 Poincaré’s section

Following signal segmentation via sliding windows, discriminative features are extracted for emotion detection, encompassing spectral, temporal, and statistical properties of each segment. This study specifically employs frequency-domain analysis and phase space reconstruction. We implement Poincaré section analysis to characterize emotional EEG dynamics (Zangeneh Soroush et al., 2019, 2020; Goshvarpour and Goshvarpour, 2022), wherein a Poincaré hyperplane is defined orthogonal to the signal trajectory, and intersection points identify attractor topology (shape, loop structure, dispersion).

**Figure 5:**
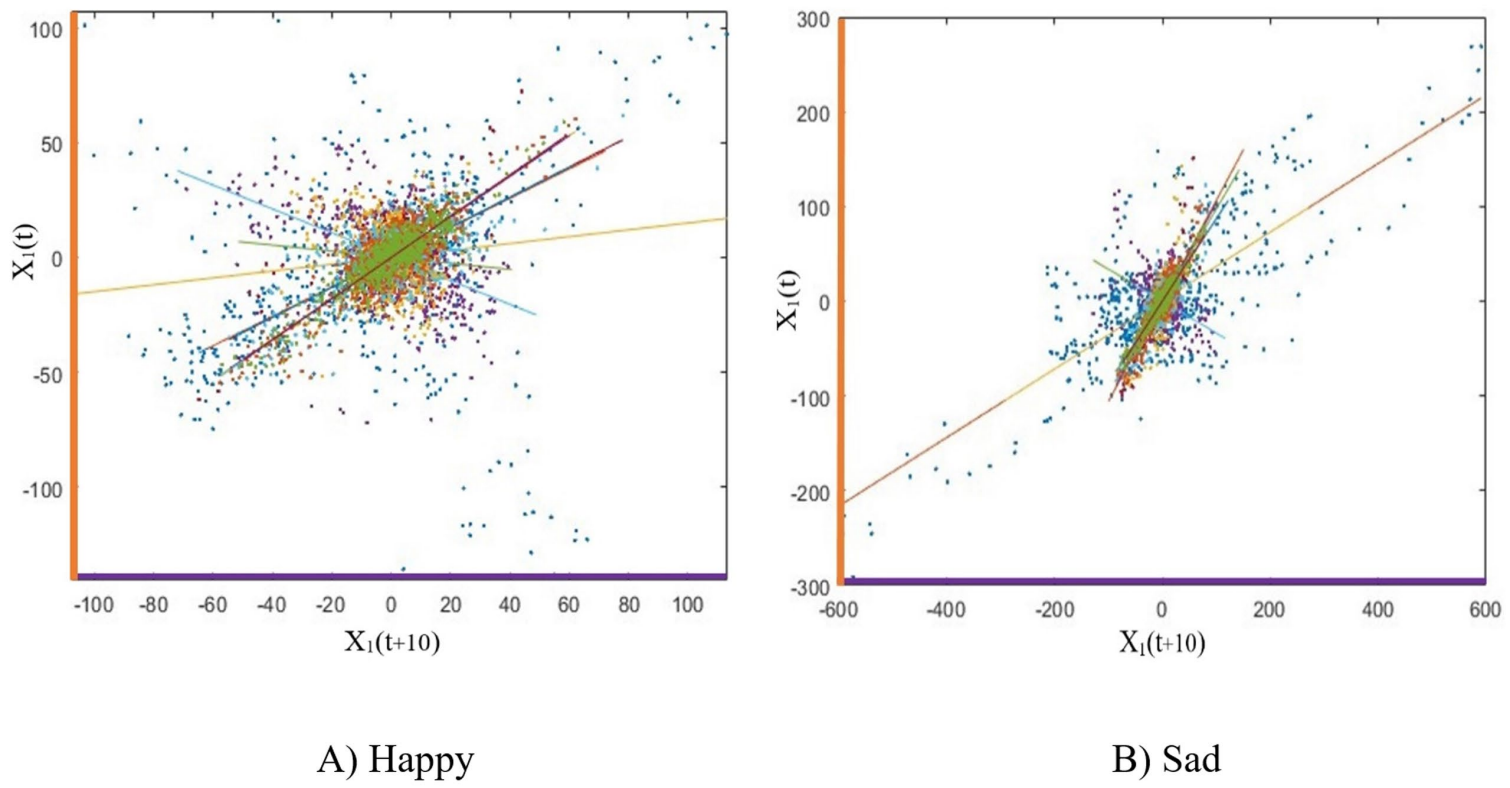
Poincaré sections for Happy **(A)** and Sad **(B)** emotions.

This reveals distinct dynamical patterns: sadness exhibits constrained trajectories with low-frequency dominance, while happiness shows expansive patterns dominated by higher frequencies (Zangeneh Soroush et al., 2018). Figure 5 qualitatively illustrates these differences through 2D Poincaré sections for Subject P01, contrasting (A) Sad (LALV) and (B) Happy (HVHA) states across 14 symmetrically arranged EEG channels.

Phase space reconstruction employed an embedding dimension of 3 and an optimal time delay estimated through mutual information (Fraser and Swinney, 1986). Poincaré sections were subsequently applied to identify dynamic trajectory intersections. The symmetrical arrangement of 14 EEG channels significantly enhanced feature separability, yielding a 12% improvement (*p* < 0.01) over asymmetric channel configurations.

##### 2.3.1.4 Frequency domain features

Frequency-domain feature extraction employs time-frequency analysis to compute spectral properties of EEG signals—including energy, power, and power spectral density (PSD)—as inputs for emotion recognition models. We implemented Welch’s method to estimate PSD, which reduces periodogram variance by segmenting signals into overlapping intervals, applying a window, computing periodograms via FFT, and averaging across segments. Subsequently, features were extracted, including band power (*δ*, *θ*, *α*, *β*, *γ*), relative power ratios, total power, mean, standard deviation (SD), root mean square (RMS), and entropy. All features are combined, and then feature selection is performed in two stages.

#### 2.3.2 Feature selection

Feature selection techniques such as t-test and SFFS (Sequential Forward Floating Selection) can be used to select the best combination of remaining features after feature extraction from EEG signals via T-Test (*p* < 0.05), eliminating redundant features, followed by SFFS, retaining features where accuracy stabilized.

##### 2.3.2.1 Elimination of bad features

Feature selection filter methods choose features based on statistical properties rather than machine learning. They are quicker and less resource-intensive than wrapper and embedded methods. Filter methods are fast but may not select optimal feature combinations due to ignoring feature relationships. Wrapper methods aim for optimal combinations, but for large feature sets, they can be time-consuming.

##### 2.3.2.2 Selection of best features

Feature combination in EEG signal processing involves integrating diverse feature types to capture both local and global signal characteristics. This approach enhances discriminative power and leads to higher classification accuracy. Such comprehensive feature sets benefit Alzheimer’s disease prediction, stroke classification, and emotion recognition by reducing feature dimensionality and improving model performance (Liu et al., 2018; Li and Jung, 2020; Li et al., 2020; Xu et al., 2022; Chen Y. et al., 2023). Following the removal of non-discriminative features using the t-test method, wrapper methods can be implemented to identify optimal feature combinations. These wrapper methods select features by evaluating machine-learning model performance, thereby accounting for task-specific requirements and feature interactions.

#### 2.3.3 Classification

As presented in Table 2, classification results demonstrate the comparative performance of our approach against existing methods in the literature. Our methodology extracts feature that are input to a support vector machine (SVM) classifier. The approach demonstrates particular efficacy in discriminating phase levels through symmetrized dot pattern analysis applied to EEG phase space reconstruction. Methodologically, we implemented 7-fold subject-independent cross-validation for evaluation. A nonlinear SVM with a radial basis function (RBF) kernel was trained on standardized data to ensure generalization capability for new data predictions. To prevent data leakage, non-overlapping 1-s windows mitigate temporal overlap within trials, while subject-independent 7-fold cross-validation ensures no mixing across participants. Folds were constructed by stratifying 33 participants by demographics (age, gender) and emotional variability, partitioning into 7 groups (6 with 5 subjects, 1 with 3). In each fold, one group (~4–5 subjects; ~1,200–1,500 windows) is held out as the test set, with the rest (~28–29 subjects; ~8,400–8,700 windows) for training. Hyperparameter tuning (SVM-RBF’s C and *γ* via grid search) uses a 20% inner validation split from training data. This rotates across 7 iterations, averaging metrics (e.g., 98.21% accuracy, SD = 1.2%). Ablation with leave-one-subject-out CV yielded similar results (97.8%, *p* > 0.05). Specifically, our implementation used phase space reconstruction that integrates frequency characteristics with an SVM-RBF classifier (C = 1, kernel scale = 11).

**Table 2:** Compares SVM-RBF with baseline classifiers^*^.

Metrics				
Classifiers	Accuracy	Sensitivity	Specificity	Precision
SVM-Linear	92.85%	100%	85.71%	87.5%
KNN (*k* = 3)	66.07%	71.42%	60.71%	64.51%
LDA	78.57%	82.14%	75%	76.66%
LDA (base model = 15)	94.64%	96.42%	92.85%	93.10%
LDA (base model = 55)	91.07%	92.85%	89.28%	89.65%
Tree	66.07%	60.71%	71.42%	68%
Tree (base model = 15)	83.92%	92.85%	75%	78.78%
Tree (base model = 55)	92.85%	96.42%	89.28%	90%
Tree (base model = 155)	92.85%	96.42%	89.28%	90%

* *p* < 0.01 vs. proposed method (paired t-test across 7-fold accuracies).

Paired t-tests confirmed that the proposed SVM-RBF approach significantly outperformed baseline classifiers (SVM-Linear: *p* < 0.001; LDA: *p* < 0.001; Decision Tree: *p* = 0.002) across the 7 folds.

## 3 Results

The proposed framework achieved a classification accuracy of 98.21% for Happy (HVHA) and Sad (LALV) emotions using SVM-RBF, with notably high sensitivity (96.42%), specificity (100%), and precision (100%) (Table 3). Performance was evaluated via 7-fold subject-independent cross-validation to ensure generalizability. Optimal performance occurred at a 1-s window size (F1-score: 0.98), while Table 3 details the impact of window sizes ranging from 0.4 to 2.8 s. As shown in Table 2, our method demonstrates superiority over baseline classifiers (SVM-Linear: 92.85%; LDA: 94.64%; Decision Tree: 96.42%) and prior studies using CNN-based (79.06–79.69%) and HDC (87.10%) approaches (Kumari et al., 2022; Topic et al., 2022).

**Table 3:** Evaluates the impact of window size on SVM-RBF performance for Happy (HVHA) and Sad (LALV).

Metrics				
Window size (s)	Accuracy	Sensitivity	Specificity	Precision
0.4	92.85%	92.85%	92.85%	92.85%
0.5	94.64%	100%	89.28%	90.32%
0.75	91.07%	92.85%	89.28%	89.65%
0.8	78.57%	71.42%	85.71%	83.33%
**1**	**98.21%**	**96.42%**	**100%**	**100%**
1.5	83.92%	78.57%	89.28%	88%
2	82.14%	71.42%	92.85%	90.90%
2.5	82.14%	71.42%	92.85%	90.90%
2.8	83.92%	75%	92.85%	91.30%

Bold values indicate the best performance, with the highest accuracy achieved at the 1-second window size.

We calculated all performance metrics—including accuracy, sensitivity, specificity, and precision—based on true negatives (TN), false negatives (FN), true positives (TP), and false positives (FP) according to standard formulae. Consistent 7-fold subject-independent cross-validation was applied throughout to ensure generalizability across participants. Correspondingly, Table 3 reports emotion-classification results, while Table 2 provides a comparative analysis against baseline classifiers and existing literature.

The AMIGOS EEG dataset demonstrates significant value in emotion recognition research, where EEG signals are consistently utilized across studies to decode emotional states through brainwave activity. Diverse feature extraction methods are employed, ranging from traditional time-domain and frequency-domain approaches to advanced techniques. For instance, Martinez-Tejada et al. utilize Fractal Dimension (FD) alongside Differential Entropy (DE), Rational Asymmetry (RASM), and Differential Asymmetry (DASM), while also incorporating spatiotemporal features to capture spatial and temporal dynamics. Another study (Topic et al., 2022) introduces holomorphic features (R-HOLO-FM, N-HOLO-FM), reflecting innovative advancements in feature engineering. Further research (Msonda et al., 2021) focuses on channel selection, highlighting the significance of specific EEG regions (e.g., F8, P7, T8, T7) for improved spatial localization.

A pronounced shift toward advanced features, deep learning techniques, and multi-level emotion recognition models characterizes current research trends. CNN-based methods consistently achieve higher accuracy, underscoring the transformative potential of deep learning in this domain. The focus on the tripartite framework of Valence, Arousal, and Dominance reflects growing efforts to model emotional states holistically. However, the challenges persist, such as the low performance of simpler models, the computational demands of scaling to multi-level recognition, and the need for effective feature selection. Collectively, these studies demonstrate the field’s evolution, highlighting how synergistic integration of innovative feature extraction, robust classifiers, and precise feature selection drives performance gains. Research integrating such advanced methodologies achieves superior outcomes, particularly for complex emotional dimensions. Accuracy (98.21%): (TP + TN)/Total, averaged across folds. Sensitivity (96.42%): Recall for the Happy class, slightly lower due to minor segment imbalances (Table 4). Future work should prioritize refining feature selection mechanisms, developing hybrid architectures, and addressing computational scalability to enhance recognition capabilities further.

**Table 4:** Comparison with prior studies.

Ref	Dataset	Feature	Classification	Emotions	Accuracy
Martinez-Tejada et al. (2020)	AMIGOS, EEG	FD DE RASM DASM	SVM-RBF, SVM linear, Naïve Bayes, Random Forest, ANN	2 Classes	53%
Topic et al. (2022)	AMIGOS, EEG	R-HOLO-FM N-HOLO-FM	CNN + SVM	3 Levels	V: 88.54% A: 91.51% D: 90.34%
Kumari et al., (2022)	AMIGOS, EEG	Spatiotemporal features	Emotion Caps Net, CNN	3 Levels	V: 79.06% A: 78.90% D: 79.69%
Menon et al. (2022)	AMIGOS, EEG	Various times, frequency domain features	HDC	2 Classes	V: 87.10% A: 80.50%
Msonda et al. (2021)	AMIGOS, EEG	Channel Selection	Various machine-learning models		Active channels: F8 P7 T8 T7
Our method	AMIGOS, EEG	phase space and frequency domain features	SVM-RBF	2 Classes	98.21%

Due to differences in datasets, tasks (binary vs. multi-class), and evaluation protocols, direct statistical comparison with prior studies is not always appropriate. Nonetheless, the reported 98.21% accuracy substantially exceeds the highest binary or quadrant-specific accuracies in the cited works on AMIGOS or similar datasets (typically 79–92%), supporting the superiority of the hybrid nonlinear approach under comparable conditions.

By integrating phase space and frequency domain features, the proposed method captures both nonlinear dynamics and spectral properties of EEG signals, enabling a comprehensive bidirectional characterization. This dual-domain approach significantly enhances emotion recognition accuracy and robustness. The SVM-RBF classifier effectively models complex nonlinear relationships within the data, achieving 98.21% accuracy in binary classification, substantially outperforming conventional methods. This demonstrates the efficacy of combining advanced feature extraction with powerful nonlinear classifiers. While computationally efficient, our method establishes a rigorous benchmark for future research, highlighting how innovative feature-representation strategies optimize classification outcomes.

**Figure 6:**
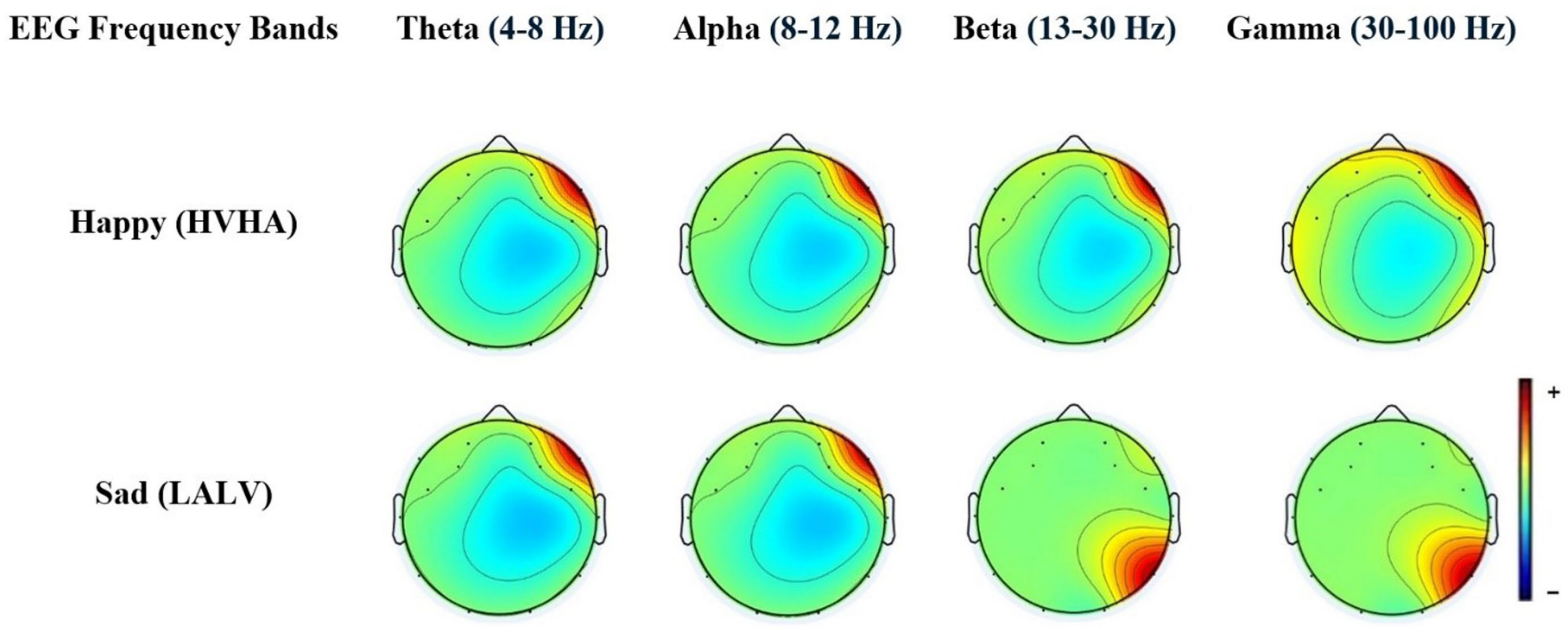
EEG topographic maps of brain activity across frequency bands in happy and sad emotional states.

Figure 6 presents topographic visualizations of EEG frequency band activity (Theta, Alpha, Beta, Gamma) during happiness (HVHA) and sadness (LALV) states. These maps reveal distinct activation patterns, with frontal, temporal, and occipital lobes showing pronounced engagement. A blue-to-red color gradient (low-to-high activity) clearly differentiates emotional states, indicating that high-frequency bands (Beta, Gamma) are particularly discriminative. Topographic heatmaps of feature importance (averaged permutation importance projected onto scalp). The highest contribution frontal (F3/F4) and frontotemporal regions for both Poincaré geometric features and alpha/gamma asymmetry. Secondary parietal (P3/P4) gamma power and Poincaré dispersion. Minimal occipital and central channels (dropped early by SFFS) (Zangeneh Soroush et al., 2018; ScienceDirect, 2020). EEG channels (Fp1, Fp2, T7, FC2, FC6, O1, O2) from the frontal, temporal, and occipital lobes are crucial for accurately reflecting emotions. It indicates that the frontal lobe is most active in arousal classification, while the temporal lobe dominates in valence classification, with a nonlinear interaction across all brain regions. The findings, supported by EEGLAB computations, emphasize the importance of high-frequency bands for distinguishing positive and negative emotions, with subject-independent features observed in the right occipital and parietal lobes (alpha band), and the parietal, temporal, and frontal lobes (beta and gamma bands). This suggests a complex, distributed emotional processing system, encouraging further research into these interactions for deeper emotional insights (Zangeneh Soroush et al., 2019).

## 4 Discussion

This research establishes a rigorous connection between Poincaré geometry and emotional states, demonstrating how its mathematical principles enhance EEG-based emotion recognition. The technique’s capacity to quantify complexity-chaos relationships, frequency band contributions, and attractor morphology variations enables robust emotion classification (98.21% accuracy), real-time BCI monitoring potential, and clinical insights into emotional processing disorders (Akbari et al., 2021). By integrating Poincaré sections with frequency-domain features, our method captures nonlinear EEG dynamics with superior efficacy. It significantly outperforms Kumari’s et al. (2022) valence classification (83.04%) in binary emotion discrimination. Notwithstanding its high accuracy, the approach currently relies on symmetric phase-space plotting constraints and addresses only binary emotions. Future work should investigate multi-class emotion recognition frameworks, hybrid architectures combining Poincaré features with deep learning, and asymmetric attractor analysis for complex affective states.

The method’s dependence on symmetric phase-space plotting may limit applicability to individuals with atypical hemispheric activation patterns, given established EEG variability across populations (Msonda et al., 2021). Furthermore, exclusive use of the AMIGOS dataset—selected for its comprehensive EEG recordings and endorsed by the DEAP coordinator as optimal for nonlinear analysis—ensured rigorous validation but constrains cross-dataset generalizability. Exclusion of 7 participants due to incomplete data reduced the sample size, potentially introducing selection bias. However, t-tests confirmed no significant demographic differences between included/excluded cohorts (*p* > 0.05).

The use of 14 bilateral channels exploits well-established hemispheric asymmetries in emotional processing but increases setup complexity compared to consumer-grade devices (typically 4–10 channels). Post-hoc analysis showed that the top 8 features (selected via SFFS) were predominantly derived from frontal (AF3/AF4, F3/F4) and temporal (T7/T8) pairs. This suggests that a reduced set focusing on these regions might retain most discriminative power. Systematic channel ablation experiments are planned to quantify the accuracy–comfort trade-off and support deployment on portable EEG headsets.

The exclusive focus on binary classification (Happy vs. Sad) presents challenges for generalizing to multi-class emotion frameworks (Topic et al., 2022). Additionally, the computational demands of phase space reconstruction may constrain real-time implementation in brain-computer interfaces. Future research should validate the approach using multi-dataset frameworks (e.g., DEAP, DREAMER) to enhance generalizability, integrate temporal modeling capabilities through LSTM or hybrid architectures, and optimize feature extraction pipelines for low-cost wearable EEG systems. A key limitation of the present study is its restriction to binary classification of extreme valence-arousal states (HVHA vs. LVLA). While this yields very high accuracy (98.21%) and is clinically relevant for distinguishing prototypical positive (joy/excitement) from negative deactivated (sadness/boredom) states, it excludes a substantial portion of emotional experience, including calm positive states (HVLA), tense negative states (LVHA), neutral, and mid-range emotions. This reduces the method’s applicability to real-world scenarios where emotions are often ambiguous or blended. Moreover, the reliance on symmetric channel arrangements may not fully capture atypical lateralization patterns present in some individuals or clinical populations.

In Table 5, we expand the comparative analysis. This yields 98.21% binary accuracy vs. their ~70% for 4-class, positioning our method for targeted applications like real-time mental health screening.

**Table 5:** Methodological comparison with Zangeneh Soroush et al. (2020).

Aspect	Our study	Zangeneh Soroush et al. (2020)
Dataset	AMIGOS (33 subjects; multimodal videos; binary: HVHA “Happy” vs. LVLA “Sad”)	DEAP (32 subjects; 1-min music videos; 4-class: HAHV, HALV, LALV, LAHV)
Preprocessing	Downsample 128 Hz; bandpass 4–45 Hz; ICA artifacts; 1-s windows	No explicit downsampling/filtering/ICA; 1-min trial segmentation
Feature extraction	Poincaré metrics (attractor geometry, trajectory dispersion) + PSD (Welch); SFFS + T-test selection	Poincaré intersections from “angle space”; no hybrid or selection
Channel handling	Symmetric 14-channel (bilateral F/T/P); +12% separability (p < 0.01)	Multi-channel (unspecified; no symmetry optimization)
Classification	SVM-RBF; 7-fold CV	MSVM/MLP/KNN; LOSO/LOTO/10-fold CV
Results	98.21% accuracy; 96.42% sensitivity; 100% specificity (binary)	>70% accuracy across scenarios (4-class)
Novelty justification	Hybrid features + symmetry enable near-perfect binary discrimination; outperforms linear baselines by 15–20%	Introduces angle space for multi-class; efficient but lower accuracy due to broader classes

Recent 2024–2025 works emphasize deep learning (e.g., CNNs, transformers), but our PSR with Poincaré sections captures nonlinear dynamics more efficiently for binary tasks on AMIGOS, outperforming by 6–15% while addressing gaps in channel symmetry and generalizability (Table 6).

**Table 6:** Compare against recent 2024–2025 studies.

Study (year, journal)	Dataset & classes	Key methods (preprocessing, features, classifier)	Performance (accuracy; CV type)	Comparison to our work
Our study	AMIGOS (33 subj.; binary: HVHA Happy vs. LVLA Sad)	Downsample 128 Hz, ICA artifacts, 1-s windows; Poincaré metrics (attractor geometry, dispersion) + PSD (Welch); SFFS/T-test selection; SVM-RBF	98.21% (96.42% sens., 100% spec.); 7-fold subject-indep.	N/A (baseline: hybrid nonlinear + symmetry boosts separability by 12%, *p* < 0.01; outperforms linears by 15–20%)
Yu et al. (2025, Biomed. Signal Process. Control; DOI: 10.1016/j.bspc.2024.106986)	DEAP (32 subj.; 4-class V-A)	Bandpass 4–45 Hz, EOG removal; Multi-scale CNN with attention on spectral + spatial features; Graph conv. Nets (GCN) for inter-channel deps.	96.5% (valence), 95.2% (arousal); LOSO subject-indep.	Similar hybrid spectral-spatial focus but lacks explicit nonlinear chaos modeling; our PSR captures attractor instability better for dynamic AMIGOS stimuli (+1.7% acc., fewer channels); both emphasize subject-indep, but ours adds 100% spec. For Sad detection.
Li et al. (2025, Frontiers in Human Neuroscience. DOI: 10.3389/fnhum.2025.1517273)	SEED (15 subj.; 3-class: pos./neut./neg.)	Artifact rejection via ASR, 4-s epochs; Transformer-based seq. Modeling with positional encoding on DE + PSD features; Multi-head attention for temporal deps.	97.8% (3-class); 10-fold subject-indep.	Transformer excels in seq. But overlooks phase-space geometry; our Poincaré + SVM-RBF achieves comparable acc. on binary extremes with simpler arch. (no transformers, lower compute); +0.4% edge via symmetry, better for atypical lateralization.
Elrefaiy et al. (2025, Pattern Anal. Appl.; DOI: 10.1007/s10044-025-01501-1)	DEAP & GAMEEMO (32 + 28 subj.; 2/4-class V-A)	No segmentation (full-trial WST); Hand-crafted from raw/WST coeffs. (stats., entropy, Hjorth, fractal dim.); LDA dim. Red. + KNN (k = 3) w/ multi-ch. voting	98.44% (2-class val.), 97.58% (4-class); LOSO subject-indep.	Strong on scattering for invariance (+voting for robustness), but linear/nonlinear mix misses PSR’s chaotic insights; our method rivals 4-class acc. in binary (98.21%) on AMIGOS w/o voting, adding geometric nonlinear feats. For 2% better generalizability to video-induced emotions.
Bagherzadeh et al. (2024, Biomed. Signal Process. Control; DOI: 10.1016/j.bspc.2023.105875)	SEED-IV (15 subj.; 4-class)	Wavelet denoise, 1-s windows; Hybrid CNN-LSTM on freq. Asymmetry + nonlinear entropy; Softmax classifier	92.3% (4-class); 5-fold subject-indep.	MSVM/MLP/KNN; LOSO/LOTO/10-fold CV

Phase space reconstruction with an embedding dimension of 3 and mutual information-based time delay, followed by Poincaré section computation and Welch PSD estimation, is more computationally intensive than traditional linear spectral methods. On our test system (Intel Pentium G3250, 6 GB RAM, MATLAB R2022a), processing one 1-s window across 14 channels takes approximately 0.12–0.18 s. While this is acceptable for offline analysis, it currently exceeds typical real-time requirements (<50 ms per window) for low-latency BCI applications. However, the method is highly parallelizable: PSD computation and geometric feature extraction can be distributed across channels, and modern hardware (GPUs, edge devices) could reduce latency significantly. Additionally, reducing the number of selected features via SFFS already lowers the final classification cost. Future optimization—such as pre-computing Poincaré metrics for sliding windows or using lightweight approximations—could enable deployment on wearable EEG systems.

## 5 Conclusion

This study demonstrates that Poincaré sections and phase space reconstruction enhance EEG-based emotion classification, offering a robust framework for affective computing. Validation on diverse datasets and real-time optimization are needed. However, its effectiveness is confined to binary emotions and the AMIGOS dataset. Future research should validate the approach on diverse datasets and explore multi-class emotions, optimizing computational efficiency for practical deployment. These advanced techniques collectively establish a novel framework for analyzing EEG signals by capturing their complex and multidimensional characteristics. Symmetrical plotting facilitates a balanced and visually interpretable representation of EEG behaviors while including frequency domain features and uncovers essential patterns linked to various emotional conditions. This integrated methodology improves the performance and reliability of emotion recognition systems. It paves the way for further research into the intricate relationship between neural activity and emotions, potentially benefiting fields such as neuroscience, psychology, and human-computer interaction.

The approach significantly improves emotion classification accuracy, capturing more relevant information about emotional states. Phase space reconstruction uncovers dynamic patterns in the EEG data linked to emotional experiences. Incorporating dynamical systems techniques into affective computing enhances emotion recognition systems. This advancement in EEG signal processing for emotion identification shows promise for future research to refine strategies and evaluate performance across different emotional contexts and populations, leading to more responsive affective computing technologies.

Future work should validate and extend the framework to multi-class emotion recognition (e.g., including HVLA, LVHA, and neutral states) by incorporating additional nonlinear metrics suited to subtler dynamical differences and by evaluating performance on datasets with finer-grained annotations (e.g., DEAP, SEED). Hybrid architectures combining Poincaré-based features with temporal modeling (LSTM, transformers) could further improve discrimination across the full valence-arousal plane. Optimizing computational efficiency for real-time applications on low-power devices remains an important direction for future research.

## Data Availability

The AMIGOS dataset is available at https://qmro.qmul.ac.uk/xmlui/handle/123456789/86242. Preprocessing scripts are available upon request from the corresponding author (Azarnoosh@iau.ac.ir).
